# Cross‐Cultural Adaptation, Reliability and Validity of the Short Version of the Parenting Styles and Dimensions Questionnaire (PSDQ) for Spanish Populations

**DOI:** 10.1111/ipd.70098

**Published:** 2026-05-08

**Authors:** María Angeles Velló‐Ribes, María Dolores Casaña‐Ruiz, Ana Amparo Zaragoza‐Fernández, Juan Ignacio Aura‐Tormos, Jose María Montiel‐Company, Montserrat Catalá‐Pizarro

**Affiliations:** ^1^ Department of Stomatology, Faculty of Medicine and Dentistry University of Valencia Valencia Spain

**Keywords:** cross‐cultural adaptation, parenting styles, pediatric dentistry, PSDQ, psychometric validation, Spanish population

## Abstract

**Objective:**

To translate, culturally adapt, and validate the short version of the Parenting Styles and Dimensions Questionnaire for use in Spanish populations.

**Methods:**

A cross‐sectional study was conducted with parents of children aged 3–14 years attending private dental clinics, primary healthcare centers, and a university dental clinic. The adaptation process included forward translation, reconciliation, back‐translation, expert review, and pilot testing. Psychometric evaluation of 334 valid responses was performed. Exploratory factor analyses examined the seven parenting dimensions and Baumrind's three parenting styles, and internal consistency was assessed using Cronbach's alpha.

**Results:**

The Spanish version showed satisfactory structural validity, replicating the three parenting styles (authoritative, authoritarian, permissive) and a coherent seven‐dimension structure after removing three items (25, 26, 28) to improve reliability. Subscale internal consistency was acceptable (*α* = 0.84 authoritative; 0.74 authoritarian; 0.63 permissive). The authoritative style was most prevalent (*M* = 4.35, SD = 0.45). The final instrument included 29 items and showed improved reliability (*α* = 0.548).

**Conclusions:**

The short PSDQ was successfully translated and culturally adapted for Spanish‐speaking parents, demonstrating adequate validity and reliability. The validated instrument provides a practical and culturally appropriate tool for assessing parenting behaviors, with applications in research, clinical practice, and pediatric dentistry.

## Why This Paper Is Important to Pediatric Dentists

- Offers a validated, culturally adapted tool to assess parenting behaviors in Spanish‐speaking families.
- Helps clinicians better understand family dynamics that may influence a child's oral health and behavior in the dental setting.
- Supports more targeted prevention and intervention strategies by integrating parenting assessment into pediatric dental care.

## 1 Introduction

Behavior encompasses actions, reactions, and responses to environmental stimuli and social interactions. In children, behavior is influenced by various factors including parental guidance, cultural background, fear, anxiety, cognitive development, and temperament [1, 2, 3].

Early childhood represents a critical period in which the foundations for health‐related behaviors are established. During this stage, parents play a pivotal role in shaping their children's behaviors and attitudes through modeling, parenting practices, and direct interactions. Research suggests that parenting behavior significantly affects children's health and developmental outcomes [1, 4, 5].

Parenting style can be categorized in two primary dimensions: parental control, which encompasses discipline, supervision, and autonomy granting, and parental warmth, which includes affection, acceptance, and engagement with the child. These dimensions define distinct parenting styles [6]. Parenting style is a complex construct that integrates attitudes, beliefs, and behaviors to create an emotional environment that influences children's behavioral responses and coping mechanisms. In pediatric dentistry, parental influence plays a crucial role in determining a child's behavior during dental visits, which can ultimately impact treatment success [1, 2, 7].

The Parenting Styles and Dimensions Questionnaire (PSDQ) [8] was the first widely recognized instrument used to assess parenting styles. It was based on Baumrind's classification of three major parenting styles: authoritative, authoritarian, and permissive [9]. Authoritative or democratic parenting is characterized by high levels of warmth and control, where parents encourage autonomy while establishing clear and reasonable limits. They employ positive discipline strategies, fostering independence, exploration, and emotional regulation in children. In contrast, authoritarian parenting is marked by high control but low warmth. These parents impose strict rules and frequently use punitive discipline. Their approach is predominantly unidirectional, offering limited responsiveness to children's emotional needs. At last, permissive parenting is defined by high warmth but low control [7]. Permissive parents provide substantial emotional support but set few boundaries, which may hinder children's ability to self‐regulate and adapt to structured environments [8, 10]. These parenting styles are summarized in Table 1, which outlines their key characteristics and associated dimensions.

**TABLE 1:** Summary of parenting styles based on the PSDQ by Robinson et al. [8].

Parenting style	Dimension	Illustrative behaviors
Authoritative	Warmth & involvement	Is affectionate, comforts the child, knows the child's friends and interests
	Reasoning & induction	Explains rules, discusses consequences, uses reasoning
	Democratic participation	Encourages child to express opinions, involves child in decision‐making
Authoritarian	Verbal hostility	Yells, argues, criticizes
	Corporal punishment	Uses physical discipline such as spanking or slapping
	Nonreasoning discipline	Enforces rules through threats or punishment without providing explanation
	Directiveness	Demands obedience, frequently scolds, issues orders without discussion
Permissive	Lack of follow‐through	Makes threats of punishment but doesn't follow through, gives in easily to child's demands
	Ignoring misbehavior	Tolerates inappropriate behavior, allows the child to interrupt or break rules
	Self‐confidence	Appears unsure or inconsistent in parenting decisions or strategies

In 2018, Oliveira et al. [6] translated and adapted a short version of the PSDQ for use in Brazil and investigated its validity and reliability. Their results demonstrated that the instrument is a reliable psychometric tool for assessing childrearing strategies according to Baumrind's model of parenting styles.

Recent trends indicate a rise in permissive parenting, coinciding with an increasing number of children who exhibit diminished self‐discipline and essential coping skills [3, 10], particularly in medical and dental settings. Pediatric dentists have noted that specific parenting styles may negatively influence children's behavior and parental acceptance of behavior management techniques in clinical practice [11, 12]. Although a validated scale exists to measure parenting styles among parents of adolescents in Spain, no equivalent tool has been adapted for younger children [13]. Thus, the objective of this study is to translate and adapt the short version of the PSDQ for use in Spain, evaluating its reliability and validity. The adapted questionnaire is expected to facilitate further research on the relationship between parenting styles and children's behavior in pediatric dental settings.

## 2 Materials and Methods

A cross‐sectional study was conducted to assess the adaptation and validation of the PSDQ in its short version for use in Spain. Participants were parents of children from 3 to 14 years old, receiving dental care at primary healthcare centers, private clinics, and a public university dental clinic.

### 2.1 Parenting Styles and Dimensions Questionnaire (PSDQ)—Short Version

For this study, a short version of the PSDQ, adapted from Oliveira et al. [6] and originally based on the 62‐item questionnaire by Robinson et al. [8], was developed and translated (Table 2). Each question was rated by participants using a 5‐point Likert scale ranging from 1 (never) to 5 (always). This format allowed for the assessment of the frequency with which parents engage in specific behaviors or adopt certain disciplinary strategies in their interactions with their children.

**TABLE 2:** Item‐by‐item comparison between the Spanish and Oliveira Short PSDQ version (based on the original full scale by Robinson et al. [8]).

Oliveira question	Robinson question	Oliveira full question	Spanish adaptation
Q1	Q21	I am responsive to child's feelings or needs	Presto atención a los sentimientos o necesidades de nuestro hijo/a
Q2	Q37	I use physical punishment as a way of disciplining our child	Utilizo el castigo físico como forma de disciplina para nuestro hijo/a
Q3	Q31	I take child's desires into account before asking child to do something	Tengo en cuenta los deseos de nuestro hijo/a antes de pedirle que haga algo
Q4	Q56	When our child asks why (he)(she) has to conform, I state: because I said so, or I am your parent, and I want you to	Cuando nuestro hijo/a pregunta por qué tiene que obedecer le contesto: “porque lo digo yo o porque soy tu padre/madre y quiero que lo hagas”
Q5	Q53	I explain how we feel about our child's good and bad behavior	Le explico a nuestro hijo/a cómo nos sentimos cuando se porta bien y cuando se porta mal
Q6	Q6	I spank when our child is disobedient	Cuando nuestro hijo/a es desobediente le doy una palmada
Q7	Q1	I encourage our child to talk about the child's troubles	Animo a nuestro hijo/a hablar de sus problemas
Q8	Q4	I find it difficult to discipline child	Me resulta difícil disciplinar a nuestro hijo/a
Q9	Q48	I encourage our child to freely express even when disagreeing with parents	Animo a nuestro hijo/a a expresarse libremente, incluso cuando no está de acuerdo con nosotros
Q10	Q10	I punish by taking privileges away from child with little if any explanation	Castigo a nuestro hijo/a quitándole privilegios, con poca o ninguna explicación
Q11	Q62	I emphasize the reasons for rules	Hago hincapié en el porqué de las normas
Q12	Q12	I give comfort and understanding when our child is upset	Cuando nuestro hijo/a está enfadado/a le doy consuelo y comprensión
Q13	Q43	I yell or shout our child when the child misbehaves	Cuando nuestro hijo/a se porta mal le grito
Q14	Q5	I give praise when our child is good	Cuando nuestro hijo/a se porta bien le felicito
Q15	Q41	I give in to our child when he/she causes a commotion about something	Cedo ante una rabieta
Q16	Q32	I explode in anger toward our child	Exploto de rabia contra nuestro hijo/a
Q17	Q34	I threaten our child with punishment more often than actually giving it	Amenazo a nuestro hijo/a con castigos que no cumplo
Q18	Q55	I take into account our child's preferences in making family plans	A la hora de hacer planes para la familia tengo en cuenta las preferencias de nuestro hijo/a
Q19	Q19	I grab our child when being disobedient	Cuando nuestro hijo/a está siendo desobediente le agarro
Q20	Q20	I state punishments to our child and does not actually do them	Impongo castigos a mi hijo/a que luego realmente no llevo a cabo
Q21	Q51	I show respect for child's opinions by encouraging child to express them	Muestro respeto por las opiniones de nuestro hijo/a animándole a expresarlas
Q22	Q22	I allow our child to give input into family rules	Permito que nuestro hijo/a colabore en la elección de las normas familiares
Q23	Q17	I scold and criticize to make our child improve	Riño a nuestro hijo/a para que mejore
Q24	Q43	I spoil our child when the child misbehaves	A menudo consiento a nuestro hijo/a
Q25	Q25	I give our child reasons why rules should be obeyed	Razono con nuestro hijo/a sobre por qué debe obedecer las normas
Q26	Q54	I use threats as punishment with little or no justification	Uso amenazas como castigo con poca o ninguna justificación
Q27	Q46	I have warm and intimate times together with our child	Tengo/dedico tiempo para mostrarle afecto y cariño a nuestro hijo/a
Q28	Q28	I punish by putting ourchild off somewhere alone with little if any explanation	Castigo a nuestro hijo/a dejándolo solo en algún sitio sin darle apenas explicaciones
Q29	Q29	I help child understand the impact of behavior by encouraging discussion of consequences	Ayudo a nuestro hijo/a entender el impacto de su comportamiento animándole a hablar de las consecuencias de sus propios actos
Q30	Q50	I scold or criticize when our child's behavior doesn't meet expectations	Regaño o critico a nuestro hijo/a cuando su comportamiento no es como esperábamos
Q31	Q58	I explain the consequences of the child's behavior	Explico a nuestro hijo/a las consecuencias que tiene el comportamiento de un niño
Q32	Q43	I slap our child when the child misbehaves	Cuando nuestro hijo/a se porta mal le doy una bofetada

### 2.2 Translation and Cross‐Cultural Adaptation

The translation and adaptation process followed the five‐step methodology previously described [14], beginning with two independent translations of the original PSDQ into Spanish. A bilingual reviewer then assessed discrepancies and resolved ambiguities to create a reconciled version, which was subsequently back translated into English by two independent native English translators. Finally, the back‐translated versions were compared to the original to ensure semantic consistency.

### 2.3 Assessment of Item Clarity and Comprehensibility

A feasibility study was conducted with 32 parents who answered the questionnaire to assess the clarity of the items using a binary scale (clear/not clear). Items requiring revision included 6.7% (items 15, 23, 27) and 3.1% (items 3, 11, 19, 20, 21, 22, 28, 31). A binomial test confirmed that none of the unclear items exceeded the 20% threshold, indicating that major revisions were unnecessary. Statistical analyses verified that the translation and adaptation maintained semantic integrity and were consistent with the original version. The final assessment determined that the translation and adaptation were valid and ready for implementation.

### 2.4 Sample Size Determination

Participants were parents of children receiving dental care at three types of healthcare settings: primary healthcare centers, private clinics, and a public university dental clinic. For the purpose of statistical comparison, participants were grouped according to the type of facility attended. To ensure statistical significance, the minimum required sample size was determined using a power analysis for an ANOVA test comparing three groups (private clinics, university dental clinics, and primary healthcare centers). The analysis assumed a small to medium effect size (Cohen's *f* = 0.3), a 95% confidence level (*α* = 0.05), and a statistical power of 80% (*β* = 0.2). Based on these parameters, the required sample size per group was approximately 110 participants, leading to a total minimum sample size of 330 participants. This allocation ensures adequate representation across different healthcare settings, enabling robust comparisons and generalizability of the findings.

Although participants were recruited from three different clinical settings to increase sample heterogeneity and external validity, the primary objective of the study was the psychometric validation of the instrument. Therefore, the analyses were conducted using the pooled sample rather than performing comparative analyses between centers.

### 2.5 Statistical Psychometric Analysis

All statistical and psychometric analyses were conducted using IBM SPSS Statistics (version 29), ensuring methodological rigor in data processing and validation procedures.

Descriptive statistics were used to summarize characteristics of the participants, reporting frequencies and percentages for categorical variables, and means and standard deviations—or medians and interquartile ranges where appropriate—for continuous variables.

To evaluate the internal structure and psychometric properties of the adapted questionnaire, two exploratory factor analyses (EFA) were conducted using principal component extraction with varimax rotation. The first EFA aimed to extract more specific parenting dimensions, yielding a seven‐factor solution that captured key behavioral subcomponents (e.g., Warmth & Involvement, Reasoning & Induction, Verbal Hostility). Factors were retained based on eigenvalues greater than 1 and the interpretability of the component structure. The second EFA tested the classical three‐factor model of parenting styles (authoritative, authoritarian, permissive), revealing a structure that closely aligned with Baumrind's theoretical framework.

Internal consistency for both the global styles and the specific dimensions was assessed using Cronbach's alpha.

### 2.6 Ethics

The study was approved by the Ethics Committee of the University of Valencia, in accordance with the Declaration of Helsinki. The parents' informed consent was requested before administering the questionnaire.

## 3 Results

The total sample consisted of 385 participants, of whom 334 valid cases (86.6%) were included in the analysis after applying filters and excluding cases with missing values on key variables. Participants were parents of children receiving dental care at three types of institutions: private clinics (*n* = 114; 34.1%), a public university dental clinic (*n* = 90; 26.9%), and primary healthcare centers (*n* = 130; 38.9%).

### 3.1 Scale Validity

To establish the validity of the scale two separate analyses were conducted. The first focused on a more detailed analysis aimed to extract seven specific dimensions grounded in both theoretical constructs and prior empirical categorizations (Table 3). Factor retention was determined using eigenvalues greater than 1 and inspection of the scree plot, which supported the retention of a seven‐factor solution consistent with the theoretical structure of the instrument.

**TABLE 3:** Factorial components by dimensions.

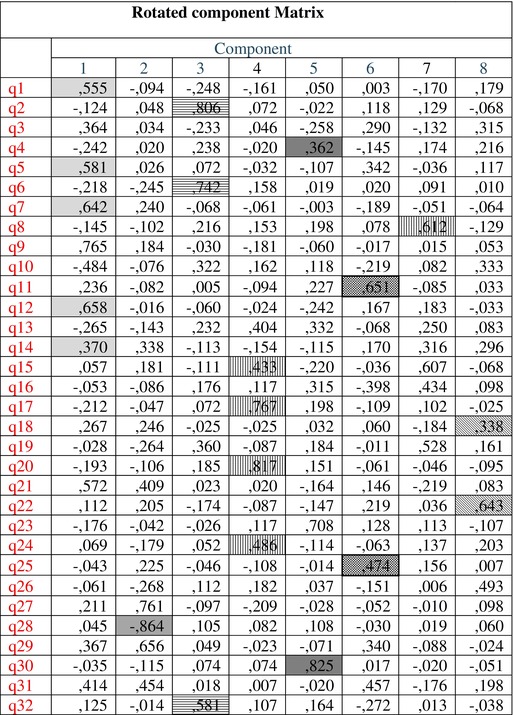

*Note:* 1: Warmth and involvement; 2: Nonreasoning, punitive strategies/directiveness; 3: Corporal punishment; 4: Lack of follow through; 5: Verbal hostility; 6: Reasoning/induction; 7: Ignoring misbehavior; 8: Democratic participation/good reasoning. The shaded cells in the rotated component matrix indicate the most relevant factor loadings for each item. Specifically, they highlight the highest loading value in each row, showing the component with which the item is most strongly associated. In some cases, shading may also be applied to loadings that exceed a predefined threshold (e.g., 0.40 or 0.50), depending on the study criteria. This visual aid facilitates the interpretation of the factor structure by allowing researchers to quickly identify the primary dimension to which each item belongs, without needing to compare all numerical values across components.

The second, three‐factor solution to validate the classical parenting style model (Table 4). In both cases, principal component extraction with varimax rotation was applied, and the resulting component structures were interpreted in the context of existing literature and established conceptual frameworks. After examining in Table 5 the factor loading matrix and the conceptual interpretation of the factors, it was decided to merge factors 4 (Lack of Follow Through) and 7 (Ignoring misbehavior) due to their high conceptual and empirical overlap. This resulted in a final structure of seven dimensions (Table 7). The matrix presented in Table 6 reveals a relatively clean factor structure where most items display their highest loading on their theoretically expected component. The 32 items were categorized into three parenting styles and seven different dimensions (Table 5).

**TABLE 4:** Factorial components by parenting style.

	Component		
	Authoritative	Authoritarian	Permissive
q1	0.525	−0.302	−0.147
q2	−0.051	0.589	0.098
q3	0.519	−0.245	0.031
q4	−0.225	0.380	−0.001
q5	0.672	−0.034	0.040
q6	−0.158	0.604	−0.212
q7	0.502	−0.138	0.174
q8	−0.128	0.561	−0.043
q9	0.711	−0.151	0.140
q10	−0.405	0.425	−0.101
q11	0.380	−0.014	−0.011
q12	0.671	−0.076	−0.006
q13	−0.263	0.601	−0.147
q14	0.505	−0.014	0.343
q15	0.076	0.354	0.203
q16	−0.149	0.496	−0.133
q17	−0.262	0.552	−0.072
q18	0.343	−0.076	0.201
q19	0.038	0.519	−0.236
q20	−0.251	0.552	−0.126
q21	0.596	−0.152	0.379
q22	0.369	−0.105	0.191
q23	−0.241	0.326	−0.030
q24	0.115	0.330	−0.203
q25	0.119	−0.011	0.306
q26	0.034	0.245	−0.319
q27	0.217	−0.182	0.726
q28	0.025	0.164	−0.863
q29	0.440	−0.071	0.674
q30	−0.151	0.343	−0.139
q31	0.564	−0.071	0.467
q32	0.023	0.465	−0.056

**TABLE 5:** Parenting styles and item distribution.

Parenting style	Dimension	Item numbers
Authoritative	Warmth & involvement	Q1, Q7, Q12, Q14, Q27
	Reasoning & induction	Q5, Q11, Q25, Q29, Q31
	Democratic participation	Q3, Q9, Q18, Q21, Q22
Authoritarian	Corporal punishment	Q2, Q6, Q19, Q32
	Non‐reasoning, punitive strategies & directiveness	Q4, Q10, Q26, Q28, Q23, Q30
	Verbal hostility	Q13, Q16
Permissive	Ignoring misbehavior, lack of follow‐through & self‐confidence	Q8, Q15, Q17, Q20, Q24

**TABLE 6:** Item‐total statistics and Cronbach's alpha if item deleted.

	Scale mean if item deleted	Scale variance if item deleted	Corrected item‐total correlation	Cronbach's Alfa if item deleted
q1	94.92	55.681	0.054	0.466
q2	98.25	53.626	0.213	0.448
q3	95.85	55.029	0.063	0.465
q4	97.55	54.266	0.115	0.459
q5	95.38	53.527	0.192	0.449
q6	98.21	54.056	0.158	0.454
q7	94.97	55.459	0.076	0.464
q8	97.27	53.573	0.166	0.452
q9	95.17	53.878	0.165	0.453
q10	97.99	55.258	0.033	0.469
q11	95.31	54.130	0.159	0.454
q12	95.36	54.111	0.144	0.456
q13	97.00	53.856	0.177	0.452
q14	95.28	53.209	0.222	0.446
q15	97.78	53.886	0.163	0.453
q16	97.85	54.104	0.147	0.455
q17	97.52	53.397	0.152	0.453
q18	95.48	54.070	0.133	0.457
q19	97.88	52.974	0.216	0.445
q20	97.69	53.598	0.133	0.456
q21	95.13	54.443	0.160	0.455
q22	96.08	53.598	0.120	0.458
q23	97.31	53.892	0.094	0.462
q24	96.77	53.986	0.161	0.454
q25	95.13	49.628	0.046	0.500
q26	97.95	47.968	0.076	0.495
q27	95.26	55.531	0.014	0.472
q28	98.43	56.451	−0.059	0.480
q29	95.32	53.865	0.144	0.455
q30	96.74	53.497	0.113	0.459
q31	95.39	52.635	0.234	0.442
q32	98.60	55.377	0.167	0.460

An analysis of the distribution of general parenting styles revealed that the authoritative style was the most prevalent among participants, with a mean score of 4.35 (SD = 0.45). Scores ranged from 2.57 to 5.07, indicating a strong tendency toward this style across the sample. The authoritarian style yielded a lower mean of 1.90 (SD = 0.43), with values spanning from 1.00 to 3.55, suggesting it was less frequently endorsed. The permissive style had an intermediate representation, with a mean of 2.30 (SD = 0.56), ranging from 1.20 to 4.00. These findings indicate a clear predominance of democratic parenting practices, whereas authoritarian and permissive styles were reported to a lesser extent.

### 3.2 Scale Reliability

#### 3.2.1 Internal Consistency Coefficients

Internal reliability of the scale of Parenting Styles & Dimensions Questionnaire was tested with the technique of Cronbach alpha (Cronbach alpha: 0.462). The low value can be explained by the presence of multiple underlying dimensions within the scale. Items belonging to different dimensions tend to correlate weakly with each other, which lowers the overall Alpha.

#### 3.2.2 Results of Parenting Styles & Dimensions Questionnaire's Subscales Internal Consistency Coefficients

According to these results, the internal consistency coefficient of Items 25, 26 and 28 were found not acceptable. After the removal of these three items from the scale, the Cronbach alpha value of the scale increased from 0.462 to 0.548 (Table 7).

**TABLE 7:** Results of dimensions and parenting style subscale's internal consistency coefficient.

Dimensions	Cronbach alpha	Parenting style	Cronbach alpha
Warmth & involvement	0.613	Authoritative/democratic	0.843
Reasoning & induction	0.686		
Democratic participation	0.666		
Corporal punishment	0.641	Authoritarian	0.738
Non‐reasoning, punitive strategies & directiveness	0.557		
Verbal hostility	0.553		
Ignoring misbehavior, lack of follow‐through & self‐confidence	0.631	Permissive	0.631
Cronbach alpha scale items: 0.548			

The final instrument consisted of 29 items out of the originally proposed 32, whereas maintaining acceptable internal consistency and construct coherence.

These adjustments contribute to the reliability and replicability of the adapted questionnaire in diverse parenting contexts.

The complete list of items included in the final version of the adapted PSDQ (excluding the removed items) is provided in Appendix. Items Q1, Q3, Q5, Q7, Q9, Q11, Q12, Q14, Q18, Q21, Q22, Q27, Q29, Q31, which are consistent with the authoritative or democratic parenting style; this style emphasizes responsive and supportive parenting practices that promote psychosocial competence, autonomy, and open communication between parent and child. Items Q2, Q4, Q6, Q10, Q13, Q16, Q19, Q23, Q30, Q32 are related to authoritarian parenting style associated with coercive strategies, including physical punishment, verbal hostility, directive control, and lack of reasoning, and items Q8, Q15, Q17, Q20, Q24 are representative of permissive parenting, reflecting inconsistency in discipline, indulgence, and weak rule enforcement.

## 4 Discussion

Understanding parenting practices requires consideration of the diverse social contexts in which families are embedded. This study aimed to adapt PSDQ to the Spanish context and assess the reliability and validity of this version using a diverse and representative sample. Participants were recruited from three different types of dental care settings—private clinics, a public university dental clinic, and primary healthcare centers—ensuring the inclusion of families from varied institutional and socioeconomic environments. This heterogeneous sample strengthens the external validity of the findings and allows for a more comprehensive understanding of parenting patterns across real‐world contexts.

In our Spanish adaptation of the PSDQ, internal consistency coefficients of 0.84 for the Authoritative subscale, 0.74 for the Authoritarian subscale, and 0.63 for the Permissive subscale reflect a pattern of intermediate reliability. Consistent with previous cross‐cultural validations, Önder and Gülay [15] reported alphas of 0.84, 0.71, and 0.38 in the Turkish sample after removing five items to optimize model fit. Pedro et al. [16] achieved alphas of 0.88, 0.73, and 0.62 in the Portuguese adaptation by retaining all 32 items and permitting three cross‐loadings for items 23, 24, and 30, whereas Oliveira et al. [6] observed alphas of 0.86–0.88, 0.84, and 0.64 for the Authoritative, Authoritarian, and Permissive scales, respectively, alongside strong test–retest ICCs (≥ 0.76). Unlike the Turkish study, which deleted items, and the Portuguese study, which introduced cross‐loadings, our Spanish version removed three items (25, 26, and 28) to improve scale performance and confirmed the original three‐factor model without further modification. These comparative findings underscore the cross‐cultural stability of the Authoritative and Authoritarian dimensions and highlight the recurring need to refine the Permissive subscale for enhanced reliability.

Although the global Cronbach's alpha of the full scale was relatively low (*α* = 0.548), this finding should be interpreted considering the multidimensional structure of the PSDQ. The questionnaire is designed to measure several distinct parenting dimensions rather than a single homogeneous construct, which may reduce the overall internal consistency when all items are analyzed together. Similar patterns have been reported in other cross‐cultural adaptations of the PSDQ. For example, the Turkish version reported a Cronbach's alpha of 0.38 for the permissive subscale [15], whereas the Portuguese version also showed lower internal consistency for this dimension [16]. In addition, previous research has highlighted the complexity and variability of parenting styles and their behaviour implications in clinical contexts, supporting the conceptual diversity underlying these constructs [17]. These results suggest that lower alpha values in multidimensional parenting scales may reflect conceptual diversity rather than poor measurement quality.

Although a fourth parenting style—neglectful or uninvolved—was introduced by Maccoby and Martin in the 1980s [18], it was not included in the present study. The Parenting Styles and Dimensions Questionnaire (PSDQ) is grounded in Baumrind's original three‐style theoretical framework (authoritative, authoritarian, and permissive), which focuses on parenting behaviors involving active engagement. Furthermore, neglectful parenting is difficult to assess through self‐report measures, as it often involves a lack of awareness or acknowledgment from the parent. As such, its inclusion would require alternative observational or multi‐informant assessment methods beyond the scope of this study.

The scoring procedure for the PSDQ followed a weighted normalization approach to address the unequal distribution of items across the three parenting style dimensions. Given the differential item allocation (Authoritative: 14 items; Authoritarian: 10 items; Permissive: 5 items), a standard summative scoring method would introduce systematic bias favoring dimensions with greater item representation.

The analytical procedure comprised four sequential steps:

1. Raw score calculation

Raw scores for each parenting dimension were computed by summing individual item responses within their respective subscales:

- Authoritative raw score (ARS) = ∑(responses to items Q1, Q3, Q5, Q7, Q9, Q11, Q12, Q14, Q18, Q21, Q22, Q27, Q29, Q31).
- Authoritarian raw score (AUS) = ∑(responses to items Q2, Q4, Q6, Q10, Q13, Q16, Q19, Q23, Q30, Q32).
- Permissive raw score (PRS) = ∑(responses to items Q8, Q15, Q17, Q20, Q24).

2 Adjustment factor computation

To compensate for differential item loading, adjustment factors were calculated using the total item count (*N* = 29) as the reference standard:

- Authoritative adjustment factor (AAF) = 29/14 = 2071.
- Authoritarian adjustment factor (AUF) = 29/10 = 2900.
- Permissive adjustment factor (PAF) = 29/5 = 5800.

3 Weighted score adjustment

Adjusted scores were derived by multiplying raw scores with their corresponding adjustment factors:

- Adjusted authoritative score (AAS) = ARS × AAF.
- Adjusted authoritarian score (AAUS) = AUS × AUF.
- Adjusted permissive score (APRS) = PRS × PAF.

4 Proportional score conversion

Final proportional scores were calculated as follows:

$$P_{i}={\mathrm{AAS}}_{i}/\text{∑AAS}$$

where *P*_*i*_ represents the proportion for parenting style *i*, AAS_*i*_ is the adjusted score for style *i*, and ∑AAS is the sum of all adjusted scores.

This methodology ensures that: (a) all proportional scores sum to unity (∑ *P*_*i*_ = 1000), (b) each parenting dimension receives equivalent weighting irrespective of item quantity, and (c) results maintain comparability across different dimensional structures while preserving the theoretical integrity of Baumrind's tripartite parenting model. The weighted normalization approach addresses the psychometric challenge of unequal subscale lengths, a common issue in multidimensional instruments where theoretical constructs are represented by varying numbers of items.

Across four cross‐cultural validations of the PSDQ short form, the underlying seven‐dimension structure—comprising Warmth/Support, Regulation, Autonomy, Physical Coercion, Verbal Hostility, Punitive Strategies, and Indulgence—has been consistently observed, yet with notable cultural nuances. In the Turkish adaptation [15], the three coercive dimensions (Physical Coercion, Verbal Hostility, Punitive Strategies) clustered robustly, whereas the single Indulgence dimension exhibited weak coherence. Pedro et al. [16] in Portugal retained all seven dimensions but introduced cross‐loadings for items bridging Regulation and Verbal Hostility, reflecting Latin cultural interpretations of “criticism” as both disciplinary and supportive. Oliveira et al. [6] in Brazil confirmed the seven dimensions via confirmatory factor analysis, with particularly strong loadings on Warmth/Support and Physical Coercion, and marginal clarity for Indulgence. Our Spanish adaptation similarly replicates all seven dimensions after removing Items 25, 26, and 28 to enhance model fit; Warmth/Support and Regulation emerge as the most stable factors, whereas Indulgence again shows the lowest internal consistency. Collectively, these findings affirm the PSDQ's multidimensional integrity across cultures, whereas highlighting the need for localized refinement—especially of the Indulgence dimension—to ensure conceptual clarity and measurement precision.

Beyond the psychometric validation of the instrument, understanding how parenting styles influence children's behavior is particularly relevant in pediatric dental settings. Moreover, our findings resonate with recent literature that has examined specific parenting dimensions in relation to children's behavioral outcomes and oral health. For instance, dimensions associated with “Warmth & involvement” and “Reasoning & induction”—hallmarks of the authoritative style—have consistently been linked to more adaptive child behavior in the dental setting [2, 19]. These parents are more likely to foster emotional regulation and cooperation in their children, which may translate to greater ease during clinical procedures.

Conversely, dimensions grouped under “Verbal hostility,” “Corporal Punishment,” and “Non‐reasoning/Punitive strategies”—aligned with the authoritarian style—have been associated with increased behavioral difficulties and dental anxiety [3, 11]. These disciplinary tactics, characterized by high control and low responsiveness, may foster fear or defiance, complicating interactions in clinical environments.

In the permissive domain, dimensions such as “Lack of follow‐through,” “Ignoring misbehavior,” and “Self‐Confidence” reflect patterns of indulgence and inconsistency. These behaviors were similarly identified in Viswanath et al. [19] and Thamilvanan et al. [3], where permissive parenting was linked to higher rates of negative behavior and increased caries risk. The lack of boundaries and structure may hinder the development of self‐regulation and responsibility in children, with implications for both behavior and health outcomes.

Although our factor structure differs slightly from other models in terms of nomenclature and item distribution, the conceptual similarities validate the multidimensional structure of parenting practices and reaffirm their relevance across contexts. The consistency of these associations across independent studies highlights the practical utility of examining parenting through specific behavioral dimensions rather than broad style categories alone.

Several limitations of the present Spanish adaptation should be acknowledged. First, the sample was composed exclusively of parents whose children attended dental care settings, which may limit the generalizability of findings to broader community or clinical populations. Second, three items (25, 26, and 28) were removed to improve factorial fit; future research should examine whether rewording or replacement of these items can enhance subscale reliability. Third, we did not conduct test–retest analyses, so the temporal stability of the Spanish version remains unverified. Finally, criterion validity was not assessed against external measures of parenting behavior or child outcomes, limiting conclusions about the instrument's practical predictive utility. Addressing these issues in subsequent studies—through more diverse sampling, multi‐informant designs, longitudinal stability testing, and criterion‐related validation—will strengthen the cross‐cultural robustness of the PSDQ in Spanish‐speaking contexts.

The findings of this study confirm that the short version of the Parenting Styles and Dimensions Questionnaire (PSDQ) has been successfully adapted and validated for use in the Spanish context. The instrument demonstrates strong psychometric properties and effectively distinguishes not only between specific parenting dimensions but also between the three main parenting styles: authoritative, authoritarian, and permissive.

Importantly, this tool offers practical value for clinical and educational settings by enabling the identification of parenting profiles that may influence children's behavior, particularly in pediatric dental environments. Understanding a caregiver's predominant style allows professionals to anticipate potential challenges and adapt communication and behavior management strategies accordingly. As such, the adapted PSDQ provides a culturally relevant and reliable resource for both research and applied practice in the Spanish‐speaking population.

## Author Contributions

M.D.C.‐R., M.C.‐P., and M.A.V.‐R. conceived the ideas. J.I.A.‐T., A.A.Z.‐F., M.D.C.‐R., and M.A.V.‐R. collected the data. J.M.M.‐C. analyzed the data. M.A.V.‐R. and M.D.C.‐R. led the writing.

## Funding

The authors have nothing to report.

## Ethics Statement

This study was approved by the Ethics Committee of the University of Valencia. Ethical clearance was granted under protocol number 2024‐ODON‐3252364, ensuring that all procedures involving human participants adhered to the established ethical standards and guidelines for clinical research.

## Consent

Written informed consent was obtained from all participants prior to inclusion in the study.

## Conflicts of Interest

The authors declare no conflicts of interest.

## Data Availability

The data that support the findings of this study are available from the corresponding author upon reasonable request.

## Parenting Styles and Dimensions Questionnaire—Spanish Adaptation

**Table:**

Questions	Spanish adaptation
Q1	Presto atención a los sentimientos o necesidades de nuestro hijo/a
Q2	Utilizo el castigo físico como forma de disciplina para nuestro hijo/a
Q3	Tengo en cuenta los deseos de nuestro hijo/a antes de pedirle que haga algo
Q4	Cuando nuestro hijo/a pregunta por qué tiene que obedecer le contesto: “porque lo digo yo o porque soy tu padre/madre y quiero que lo hagas”
Q5	Le explico a nuestro hijo/a cómo nos sentimos cuando se porta bien y cuando se porta mal
Q6	Cuando nuestro hijo/a es desobediente le doy una palmada
Q7	Animo a nuestro hijo/a hablar de sus problemas
Q8	Me resulta difícil disciplinar a nuestro hijo/a
Q9	Animo a nuestro hijo/a a expresarse libremente, incluso cuando no está de acuerdo con nosotros
Q10	Castigo a nuestro hijo/a quitándole privilegios, con poca o ninguna explicación
Q11	Hago hincapié en el porqué de las normas
Q12	Cuando nuestro hijo/a está enfadado/a le doy consuelo y comprensión
Q13	Cuando nuestro hijo/a se porta mal le grito
Q14	Cuando nuestro hijo/a se porta bien le felicito
Q15	Cedo ante una rabieta
Q16	Exploto de rabia contra nuestro hijo/a
Q17	Amenazo a nuestro hijo/a con castigos que no cumplo
Q18	A la hora de hacer planes para la familia tengo en cuenta las preferencias de nuestro hijo/a
Q19	Cuando nuestro hijo/a está siendo desobediente le agarro
Q20	Impongo castigos a mi hijo/a que luego realmente no llevo a cabo
Q21	Muestro respeto por las opiniones de nuestro hijo/a animándole a expresarlas
Q22	Permito que nuestro hijo/a colabore en la elección de las normas familiares.
Q23	Riño a nuestro hijo/a para que mejore
Q24	A menudo consiento a nuestro hijo/a
Q27	Tengo/dedico tiempo para mostrarle afecto y cariño a nuestro hijo/a
Q29	Ayudo a nuestro hijo/a entender el impacto de su comportamiento animándole a hablar de las consecuencias de sus propios actos
Q30	Regaño o critico a nuestro hijo/a cuando su comportamiento no es como esperábamos
Q31	Explico a nuestro hijo/a las consecuencias que tiene el comportamiento de un niño
Q32	Cuando nuestro hijo/a se porta mal le doy una bofetada

## References

[1] P. D. Lanjekar , S. H. Joshi , P. D. Lanjekar , and V. Wagh , “The Effect of Parenting and the Parent‐Child Relationship on a Child's Cognitive Development: A Literature Review,” Cureus 14, no. 10 (2022): e30574, 10.7759/cureus.30574. 36420245 PMC9678477

[2] K. Shalini , K. S. Uloopi , C. Vinay , A. Ratnaditya , K. S. RojaRamya , and P. Chaitanya , “Impact of Parenting Style on Child's Behavior and Caries Experience in 3‐6‐Year‐Old Children: A Cross‐Sectional Study,” International Journal of Clinical Pediatric Dentistry 16, no. 2 (2023): 276–279, 10.5005/jp-journals-10005-2517. 37519952 PMC10373772

[3] S. Thamilvanan , D. Srinivasan , C. H. Benedict , and P. Balakrishnan , “Correlation of Personality, Temperament, and Behavior of Children in the Dental Environment,” International Journal of Clinical Pediatric Dentistry 17, no. 8 (2024): 907–912, 10.5005/jp-journals-10005-2867. 39372349 PMC11451869

[4] B. Sabbarwal , M. P. Puranik , and S. R. Uma , “Association Between Parental Behavior and Child's Oral Health Among 3–5‐Year‐Old Children in Bengaluru City,” International Journal of Clinical Pediatric Dentistry 13, no. 6 (2020): 677–681, 10.5005/jp-journals-10005-1856. 33976495 PMC8060931

[5] B. Buldur , “Pathways Between Parental and Individual Determinants of Dental Caries and Dental Visit Behaviours Among Children: Validation of a New Conceptual Model,” Community Dentistry and Oral Epidemiology 48, no. 4 (2020): 280–287, 10.1111/cdoe.12530. 32239726

[6] T. D. Oliveira , D. D. S. Costa , M. R. Albuquerque , L. F. Malloy‐Diniz , D. M. Miranda , and J. J. de Paula , “Cross‐Cultural Adaptation, Validity, and Reliability of the Parenting Styles and Dimensions Questionnaire—Short Version (PSDQ) for Use in Brazil,” Revista Brasileira de Psiquiatria 40, no. 4 (2018): 410–419, 10.1590/1516-4446-2017-2314. 29898189 PMC6899386

[7] A. Juneja and S. Aleem , “The Impact of Parenting Styles on Pediatric Dental Behavior and Anxiety in the Dental Operatory,” Journal of Dentistry for Children (Chicago, Ill.) 90, no. 3 (2023): 158–163. 38123925

[8] C. C. Robinson , B. Mandleco , S. F. Olsen , and C. H. Hart , “Authoritative, Authoritarian, and Permissive Parenting Practices: Development of a New Measure,” Psychological Reports 77, no. 3 (1995): 819–830, 10.2466/pr0.1995.77.3.819.

[9] D. Baumrind , “Effects of Authoritative Parental Control on Child Behavior,” Child Development 37, no. 4 (1966): 887, 10.2307/1126611.

[10] S. Kılıçkaya , N. Uçar , and M. Denizci Nazlıgül , “A Systematic Review of the Association Between Parenting Styles and Narcissism in Young Adults: From Baumrind's Perspective,” Psychological Reports 126, no. 2 (2023): 620–640, 10.1177/00332941211041010. 34404305

[11] A. K. Tsoi , S. Wilson , and S. Thikkurissy , “A Study of the Relationship of Parenting Styles, Child Temperament, and Operatory Behavior in Healthy Children,” Journal of Clinical Pediatric Dentistry 42, no. 4 (2018): 273–278, 10.17796/1053-4628-42.4.6. 29750619

[12] S. J. Quek , Y. F. Sim , B. Lai , W. Lim , and C. H. Hong , “The Effect of Parenting Styles on Enforcement of Oral Health Behaviours in Children,” European Archives of Paediatric Dentistry 22, no. 1 (2021): 83–92, 10.1007/s40368-020-00537-7. 32418053

[13] R. Bersabé , M. J. Fuentes , and E. Motrico , “Análisis Psicométrico de Dos Escalas para Evaluar Estilos Educativos Parentales,” Psicothema 13, no. 4 (2021): 678–684.

[14] V. D. Sousa and W. Rojjanasrirat , “Translation, Adaptation and Validation of Instruments or Scales for Use in Cross‐Cultural Health Care Research: A Clear and User‐Friendly Guideline: Validation of Instruments or Scales,” Journal of Evaluation in Clinical Practice 17, no. 2 (2011): 268–274, 10.1111/j.1365-2753.2010.01434.x. 20874835

[15] A. Önder and H. Gülay , “Reliability and Validity of Parenting Styles & Dimensions Questionnaire,” Procedia‐Social and Behavioral Sciences 1, no. 1 (2009): 508–514, 10.1016/j.sbspro.2009.01.092.

[16] M. F. Pedro , E. Carapito , and T. Ribeiro , “Parenting Styles and Dimensions Questionnaire—Versão Portuguesa de Autorrelato,” Psicologia 28, no. 2 (2015): 302–312, 10.1590/1678-7153.201528210.

[17] N. A. Aminabadi and R. M. Farahani , “Correlation of Parenting Style and Pediatric Behavior Guidance Strategies in the Dental Setting: Preliminary Findings,” Acta Odontologica Scandinavica 66, no. 2 (2008): 99–104, 10.1080/00016350802001322. 18446551

[18] E. E. Maccoby and J. A. Martin , “Socialization in the Context of the Family: Parent‐Child Interaction,” in Handbook of Child Psychology, vol. 4, ed. P. H. En (Wiley, 1983), 1–101.

[19] S. Viswanath , S. Asokan , P. R. Geethapriya , and K. Eswara , “Parenting Styles and Their Influence on Child's Dental Behavior and Caries Status: An Analytical Cross‐Sectional Study,” Journal of Clinical Pediatric Dentistry 44, no. 1 (2020): 8–14, 10.17796/1053-4625-44.1.2. 31995424

